# Estimating Hidden Population Sizes with Venue-based Sampling: Extensions of the Generalized Network Scale-up Estimator

**DOI:** 10.1097/EDE.0000000000001059

**Published:** 2019-09-30

**Authors:** Ashton M. Verdery, Sharon Weir, Zahra Reynolds, Grace Mulholland, Jessie K. Edwards

**Affiliations:** From the a Department of Sociology and Criminology, The Pennsylvania State University, University Park, PA; b Department of Epidemiology, The Gillings School of Global Public Health, The University of North Carolina at Chapel Hill, Chapel Hill, NC.

**Keywords:** HIV/AIDS, Key populations, Network scale-up methods, Social networks, Venue-based sampling

## Abstract

Researchers use a variety of population size estimation methods to determine the sizes of key populations at elevated risk of human immunodeficiency virus (HIV)/acquired immune deficiency syndrome (AIDS), an important step in quantifying epidemic impact, advocating for high-risk groups, and planning, implementing, and monitoring prevention, care, and treatment programs. Conventional procedures often use information about sample respondents’ social network contacts to estimate the sizes of key populations of interest. A recent study proposes a generalized network scale-up method that combines two samples—a traditional sample of the general population and a link-tracing sample of the hidden population—and produces more accurate results with fewer assumptions than conventional approaches.

**Methods:**

We extended the generalized network scale-up method from link-tracing samples to samples collected with venue-based sampling designs popular in sampling key populations at risk of HIV. Our method obviates the need for a traditional sample of the general population, as long as the size of the venue-attending population is approximately known. We tested the venue-based generalized network scale-up method in a comprehensive simulation evaluation framework.

**Results:**

The venue-based generalized network scale-up method provided accurate and efficient estimates of key population sizes, even when few members of the key population were sampled, yielding average biases below ±6% except when false-positive reporting error is high. It relies on limited assumptions and, in our tests, was robust to numerous threats to inference.

**Conclusions:**

Key population size estimation is vital to the successful implementation of efforts to combat HIV/AIDS. Venue-based network scale-up approaches offer another tool that researchers and policymakers can apply to these problems.

## Abstract 2

Supplemental Digital Content is available in the text.

Many national and international organizations identify certain groups as key populations at elevated risk of HIV infection and transmission, including men who have sex with men, female sex workers, and people who inject drugs.^1^ Key populations are crucial for epidemic surveillance and progress assessment.^2–5^ Likewise, deploying programs tailored to key populations improves response effectiveness and sustainability, but few countries have scaled up programs for these groups.^6^ Uncertainty over the sizes of key populations is one factor among several that impedes deployment of tailored responses, in part because key populations tend to be undercounted.^7^ Although quantifying epidemic impact, advocating for resources, and implementing prevention, care, and treatment programs all rely on accurate knowledge of key population sizes,^8^ methods of key population size estimation are underdeveloped. As such, new, valid, and deployable methods of estimating key population sizes are of broad interest.

A challenge in estimating key population sizes is that such groups are often “hidden populations” that cannot be surveyed with traditional approaches.^8,9^ Hidden populations lack a sampling frame, are rare in the general population, and are frequently unwilling to participate in standard survey protocols because of stigma, low trust, and desired anonymity.^10^ The difficult but important task of collecting data on key populations has necessitated the use of nontraditional survey methods,^11^ the most common of which are respondent-driven sampling and venue-based sampling.^12–16^ Respondent-driven sampling draws on ideas from social network analysis,^17^ which formally models groups of people and the direct and indirect ties between them through diverse relationships, including friendship, exchange, and acquaintance,^18^ and uses social networks to obtain referrals to sample participants. By contrast, venue-based sampling leverages the tendency of hidden population members to gather in identifiable locations for sampling.

Many samples are collected with respondent-driven and venue-based sampling, making these methods a potentially valuable resource for key population size estimation. Unfortunately, methods for doing so are limited. The primary aim of most applications of respondent-driven^19–21^ and venue-based sampling^22–24^ is surveillance-oriented—producing generalizable estimates of HIV prevalence and risk behaviors—and most methodological research on these sampling methods focuses on prevalence estimation.^25–32^ Few studies have developed key population size estimators for such samples, and almost all of those have focused on respondent-driven sampling, where researchers have used network scale-up,^33,34^ capture–recapture,^35,36^ and Bayesian approaches,^37,38^ drawing on tools of size estimation for traditional samples.^39–42^

Statistical work on population size estimation from venue-based samples is particularly limited. Most population size estimation with venue-based samples uses proportionate scaling approaches that rely on the sampled prevalence of respondents reporting key population-defining behaviors.^43–45^ For instance, one venue-based sampling method used for key population size estimates in more than a dozen countries is Priorities for Local AIDS Control Efforts (PLACE).^46^ PLACE constructs a sampling frame of venues in the study area where key population members like men who have sex with men gather, then sample them. Unlike respondent-driven sampling, which only samples key population members, PLACE’s protocols usually seek to over-sample—but not exclusively sample—key population members. As such, PLACE studies often sample only a few key population members, which limits the quality of key population size estimates that can be made using proportionate scaling approaches.

Given the challenges of key population size estimation in venue-based sampling, we asked whether size estimation methods currently used with respondent-driven sampling can be extended to these samples. To do this, we examined recent methods for hidden population size estimation with respondent-driven sampling data,^41,47^ expanded them for use with venue-based sampling and tested our proposed methods in a simulation evaluation framework.

## PREVIOUS RESEARCH

Traditional network scale-up approaches collect “aggregate relational data” by asking respondents how many people they know in different groups, with one group including the hidden population of interest and others including populations with administratively known numbers, like people named Martha or police officers.^33,48^ Note, eAppendix A (http://links.lww.com/EDE/B558) reviews key assumptions and notations. These approaches^33,49^ use an estimator that combines the total population size (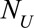) and, for each sample member, how many connections they report to members of the hidden population (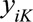) and to members of the total population (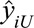).


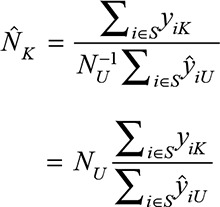
(1)

The traditional network scale-up method is powerful because it can be applied to general population samples without no requirement to survey key population members, who are likely to be hidden.^33,40,41^ This is possible because it makes several key but unrealistic assumptions that we review in eAppendix B; http://links.lww.com/EDE/B558.^33,47,50–54^

**FIGURE 1.**
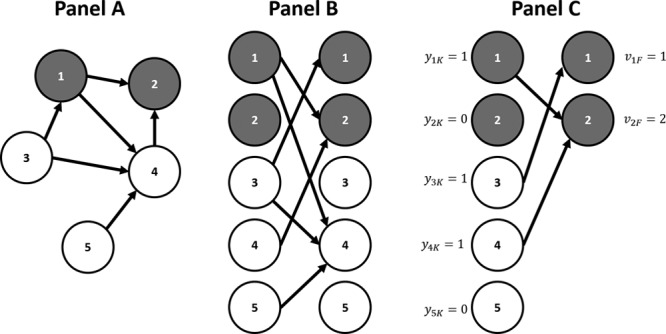
A schematic representation of the key features of the generalized network scale-up approach. Panel A shows a hypothetical social network linking persons 1–5 through directed social network ties. Key population members are shown with shaded nodes, nonkey population members are shown with unshaded nodes. Panel B represents the persons and social networks ties in panel A as a function of outgoing ties (left side) and incoming ties (right side). At the population level, the number of outgoing ties must equal the number of incoming ties. Panel C limits the outgoing and incoming ties to those that are sent to and received by key population members. The sum of ties outgoing ties to key population members 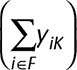 must equal the sum of the incoming ties to key population members 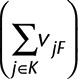. The ratio of the sum of the outgoing ties to key population members to the mean incoming ties received by key population members equals the size of the key population 
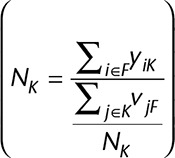
.

Recently, Feehan and Salganik^33^ introduced a new approach, the Generalized Network Scale-up Method, which replaces traditional network scale-up methods’ unrealistic assumptions with less stringent ones (see eAppendix B; http://links.lww.com/EDE/B558). This method contains two innovations. First, it uses a convenient identity in social networks: the total outgoing ties must equal the total incoming ties, a property that also holds for network subsets and means that the number of ties from any one group to any second group must equal the number of ties coming to the second group from the first. Figure 1 illustrates this identity in three panels. Panel A shows a hypothetical social network; panel B shows a different representation of the same network where each person is shown as both a sender (left) and receiver (right) of ties; and panel C shows the identity holds for network subsets.

To formally represent the ideas in Figure 1, we define a sampling frame *F* and make an important assumption that all key population members (*K*) are on the frame; that is, 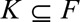 (*K* is a subset of *F*). In venue-based sampling, the frame is venue attenders and this assumption implies that all key members have nonzero probabilities of venue attendance. Let 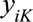 be out-reports from the *i*th member of *F* to each member of *K*, and let 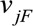 be in-reports for the *j*th member of *K* from each member of *F*. Note that we assume 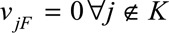 (in-reports are zero for all cases who are not key population members). In the generalized network scale-up method and our approach below, this is an assumption about accurate self-reporting of key population membership, but it only applies in one direction. Specifically, it only assumes that those who are not members of the key population do not say they are; the much more likely converse situation, wherein key population members fail to identify as such owing to stigma or other reasons, is not assumed.^33^ It is a reasonable assumption for situations where key population membership is often stigmatized, the case of interest, and because PLACE and other venue-based sampling protocols only condition sample participation and any associated incentives on venue attendance, not key population membership. If we assume no false-positive or false-negative reports, that reported ties equal real ties and all real ties are reported, assumptions we relax below, then out-reports from frame members to key population members must equal in-reports to key population members from frame members, as shown in Equation (2).


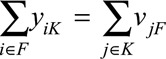
(2)

Because out-reports equal in-reports as per Equation (2), it must be the case that the total out-reports from the frame population to the key population (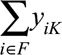) divided by the mean in-reports to the key population from the frame population (
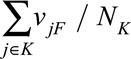
) equals the size of the key population, as shown in Equation (3). This can be seen with algebraic manipulation after substituting either of the equivalent quantities 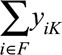 or 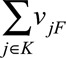 for the other.


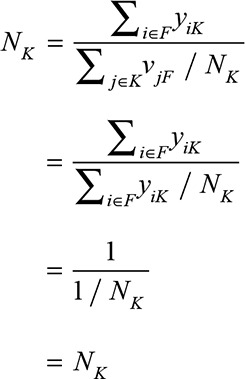
(3)

Equation (3) highlights that, if the above assumptions are met, two quantities are needed to know the size of the key population: the total number of ties from frame members to key population members 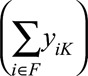 and the mean number of ties from frame members to members of the key population 
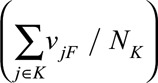
. Even in complex sampling scenarios, the insights in Equation (3) still apply if we can accurately and efficiently estimate the two quantities of interest.

Feehan and Salganik’s^33^ second innovation is to propose conducting two separate samples: one to estimate outgoing ties from the frame population to the key population (
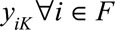
, estimating 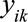 for all members of the sampling frame) and one to estimate incoming ties to the hidden population from the frame population (
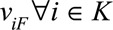
, estimating 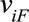 for all members of the hidden population). In their approach, the first sample must be a standard sample, i.e., a conventional probability sample (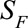), where each case is selected with potentially unequal but known inclusion probabilities from a known sampling frame that may or may not cover the total population. The second sample for the generalized network scale-up method can be focused on the hidden population and must only satisfy the criteria of being a “relative probability sample.”^33^ A sample 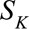 is a relative probability sample if it is selected from a potentially unknown but existing sampling frame 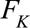 of key population members such that each person 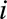 has an inclusion probability 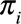 where 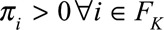 (inclusion probabilities are positive for all members of the sampling frame of key population members), but all that is observed for each sample member 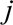 is their probability of inclusion relative to other sample members, 
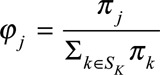
 (see eAppendix A; http://links.lww.com/EDE/B558). Relative probability samples do not require that researchers know each case’s sample inclusion probability; rather, researchers only need to know how each sample member’s inclusion probability compares to all other sample members’ inclusion probabilities. Feehan and Salganik^33^ argue that respondent-driven sampling meets the definition of a relative probability sample.

## THE GENERALIZED NETWORK SCALE-UP METHOD IN VENUE-BASED SAMPLING

There are two challenges to use generalized network scale-up method with venue-based sampling. First, individual sample inclusion probabilities are not known. Second, only one sample is obtained, and it is not a traditional probability sample; rather, it is a sample where inclusion probabilities are determined both by a multistage sampling approach and respondents’ frequency of attending venues. We develop an approach for using the generalized network scale-up method in venue-based sampling that addresses these issues.

Sample estimators for the generalized network scale-up method use out-reports to estimate the total outgoing ties from frame members to members of the key population


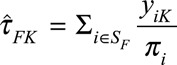
(4)

(
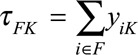
) from a conventional probability sample using the Horvitz-Thompson estimator of the population total.^33^

Equation (4) weights each sample member’s out-reports to key population members by their known sample inclusion probability, 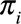. Feehan and Salganik^33^ use the relative probability sample collected with respondent-driven sampling or other link-tracing methods to estimate the average visibility of key population members. To do this, they estimate the mean number of incoming ties from frame members to key population members 
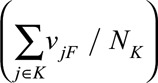
 using in-reports and a Modified Hansen-Hurwitz estimator/Generalized Horvitz-Thompson estimator of the population mean.^19^


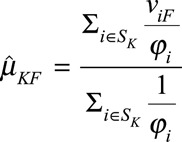
(5)

In Equation (5), 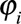 is the relative inclusion probability for the relative probability sample’s *i*th member.

Using the estimators proposed in Equations (4) and (5), the size of the hidden population can be estimated in the same fashion as achieved at the population level in Equation (3).


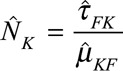
(6)

The denominator of Equation (6) can be readily estimated in venue-based sampling if relative inclusion probabilities among key population members are known. Unfortunately, the complexity of venue-based sampling precludes estimation of the numerator in Equation (6), 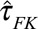, by the approach used in Equation (4) because, in this method, the sample inclusion probabilities of frame members, 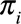, are unknown.

We argue that venue-based sampling produces relative probability samples, because the sample of venue-attending frame members, 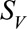, is determined by participants’ frequency of venue attendance. We can estimate relative inclusion probabilities, 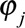, by scaling sample participants’ frequencies of venue attendance relative to each other. The challenge, then, is estimating the number of outgoing ties from frame members to members of the hidden population, 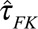, using relative probability sampling. We propose here a method for estimating this quantity in a relative probability sample. Our proposed method requires that we know the number of frame population members that are not key population members, 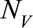. Although this assumes that researchers know the number of venue attenders and that key population members are a small fraction of them, we note that uncertainty about this number will affect results linearly such that an error of 10% in it will result in an approximate additional error of 10%. We use the same approach as described in Equation (5), but we estimate the population mean out-reports from members of 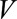 to members of 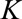 multiplied by the number of people who attend venues but are not hidden population members, 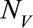. This estimator is shown in Equation (7).


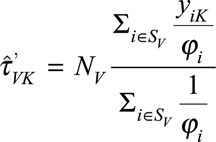
(7)

The new approach to estimating total out-reports from frame members to key population members in Equation (7) is readily adaptable to producing venue based-generalized network scale-up method estimates as shown in Equation (8).


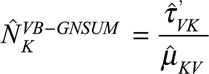
(8)

Alternative approaches to estimating the total outgoing ties from frame members to key population members are possible and produce similar results (see eAppendix C; http://links.lww.com/EDE/B558).

## DATA REQUIRED FOR VENUE-BASED–GENERALIZED NETWORK SCALE-UP METHOD

To implement the venue-based–generalized network scale-up method, we suggest that researchers using venue-based sampling ask questions that enable estimation of the outgoing ties from frame members to key population members and of the incoming ties to key population members from frame members. To take an example from a PLACE survey that focuses on sampling respondents at bars and clubs, for instance, researchers interested in estimating the number of men who have sex with men could ask all respondents the following two questions: (1) “Think of all the people you know in this district whom you have talked to in the past 4 weeks. You know them and they know you. Of those people you know, how many go out to bars and clubs?”, and (2) “Some men have sex with other men. Think of all the men who have sex with men whom you know in this district, those whom you have talked to in the past 4 weeks. You know these men and they know you. Of those men you know who have sex with other men, how many go out to bars and clubs?” Researchers should also ask respondents (3) their status in the key population of interest (whether they are men who have sex with men in this example), and (4) how frequently they attend venues and other weighting questions typically used in venue-based sampling studies.^46^ Subtracting responses to question 2 from responses to question 1 among those who are members of the key population as a measure of 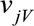, and using responses to question 2 among those who are not members of the key population as a measure of 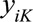, estimates of the size of the venue-attending population overall as a proxy for 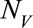, and each respondents’ frequency of attending venues and other relevant weighting variables to measure 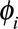, researchers can calculate the venue-based–generalized network scale-up method estimate of key population size. Additional questions suggested in network scale-up research^33,34^ can help to improve estimates of 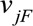 and 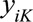.

## METHODS AND MEASURES

We tested the effectiveness of 
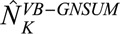
 for estimating the sizes of key populations through a series of simulations that selectively varied relevant features of the population and sampling. We aimed to assess the validity and efficiency of the venue-based–generalized network scale-up method under different situations that researchers might encounter in the field. The essence of our approach is that we generate synthetic populations with parameterized features—including size, composition, the social network linking its members together, each person’s frequency of venue attendance, and rates of falsely reporting ties to key population members—then we simulate nonrandom samples of people from these populations in line with venue-based sampling protocols. We then apply the venue-based–generalized network scale-up method to these samples to learn how different features of the population and sampling design might influence the quality of estimates produced by this method, assessing both their biasedness and their variability from sample to sample. Our simulations focused on estimator performance under four conditions that previous study has not explored: “Test A” as key population size varies; “Test B” as the sample size of key population members varies; “Test C” under different scenarios governing the sampling probabilities of members of 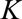 and 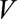; “Test D” as more false-positive ties are reported. Full simulation details are available in eAppendix D; http://links.lww.com/EDE/B558; code is available in eAppendix F; http://links.lww.com/EDE/B557.

## SIMULATION RESULTS

**FIGURE 2.**
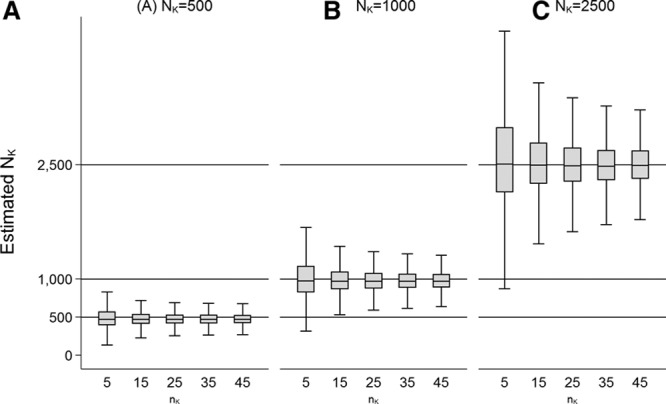
Box and whisker plots of results of test A, which varies the size of the key population to be estimated (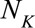), and test B, which varies the size of the sample collected from key population members (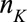). Note that outlier values are not shown.

Figure 2 shows box and whisker plots of the results of tests A and B. In these tests, we varied key population size (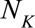) and the number of key population members sampled (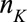); we held numbers of nonkey population members sampled (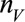) and on the sampling frame (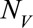) constant. The graph’s three panels are arranged in ascending order of key population size (test A), while each panel’s *x* axis is arranged in ascending order of the sample size of key population members (test B). In general, 
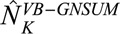
 produces adequate estimates, as either 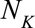
or 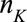 varies.

Across the range of values of test A, we found that a measure of relative bias that allows for comparisons across the population sizes (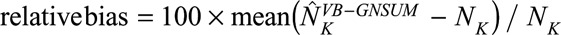) is generally low. For instance, when the sample size was 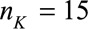, the 100,000 samples conducted at 
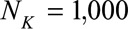
 had an average relative bias of −0.6% (undercounting the total by approximately six people, on average). Table 1 shows relative bias for all combinations in tests A–D. The largest relative bias occurred when 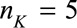 and 
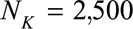
, where 
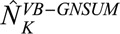
on average overcounted the key population size by 150 people (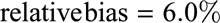).

**TABLE 1.**
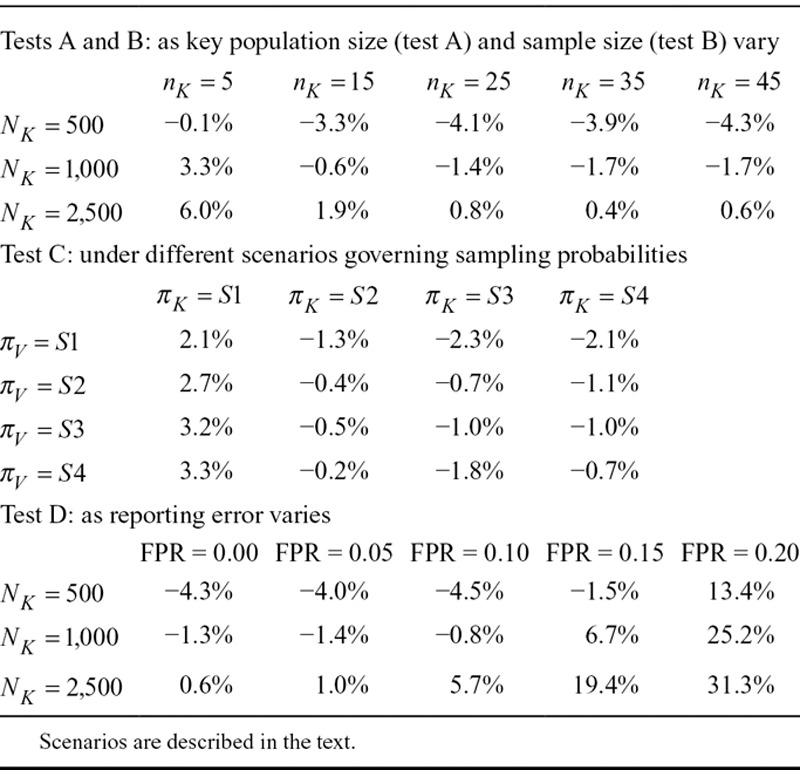
Relative Bias for Venue-based Generalized Network Scale-up Method Estimator in Each Combined Scenario of Tests A–D, Which Vary the Size of the Key Population (Test A), the Sample Size (Test B), the Sampling Probabilities (Test C), and the Reporting Error (Test D)

Regarding test B, the key finding was that the venue-based–generalized network scale-up method yielded approximately unbiased estimates with low variability even when few key population members were sampled. We measured the method’s variability from sample to sample in terms of relative standard error, which expresses the standard deviation of the distribution of mean estimates within a given combination of 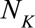 and 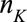 across the 100 simulated networks and 1,000 simulated samples per network as a percentage of the target population size: 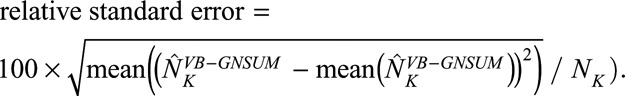 Sampling additional key population members quickly yielded declining returns in relative standard errors. For instance, when 
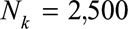
, the relative standard error declines from 31.4% when 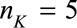 to 17.8% when 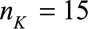 to 13.5% when 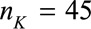; behavior is comparable at other population sizes. Table 2 shows relative standard errors for 
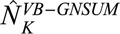
 in all tests. Although a larger sample is better, these findings suggest that the venue-based–generalized network scale-up method produces reliable size estimates even when samples contain few key population members (about 25 or so).

**TABLE 2.**
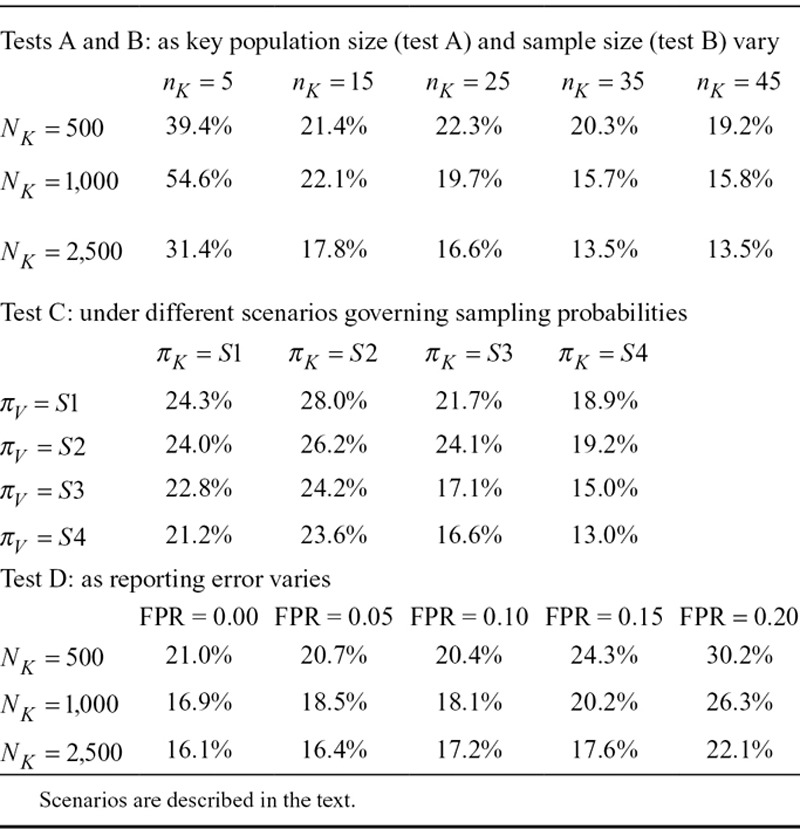
Relative Standard Errors for the Venue-based Generalized Network Scale-up Method Estimator in Each Combined Scenario of Tests A–D, Which Vary the Size of the Key Population (Test A), the Sample Size (Test B), the Sampling Probabilities (Test C), and the Reporting Error (Test D)

**FIGURE 3.**
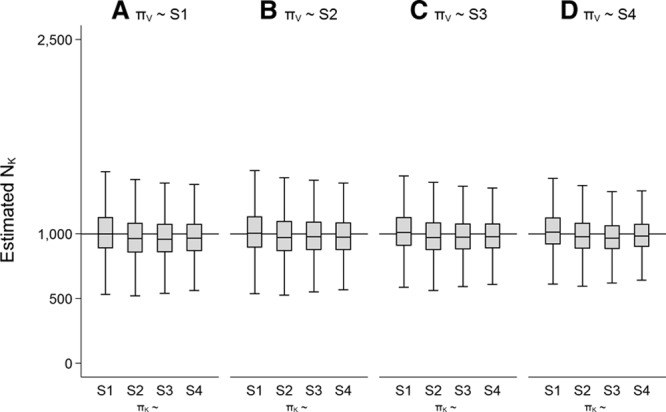
Box and whisker plots of results of test C, which explores different distributions of sample inclusion probabilities both for members of *V* and members of *K*. S1, S2, S3, and S4 refer to the scenarios described in the section discussing test C. Note that outlier values are not shown.

Figure 3 presents test C’s results, where we explored different distributions of sample inclusion probabilities while fixing key population size at 
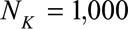
 and the size of the sample of key population members at 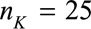. *Y* axis labeling is the same as in Figure 2 for comparability. The graph’s four panels show different distributions of sample inclusion probabilities for frame members who are not members of the key population (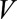). Within each panel, four distributions of sample inclusion probabilities for key population members (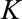) are arrayed along the *x* axis. Across all 16 combinations, the results are encouraging. At no point did relative bias exceeds ±3.3%, and relative standard errors were all less than 30.0%, although some combinations of sampling distributions led to lower standard errors than others. These results indicate that venue-based–generalized network scale-up method is robust to different distributions of sample inclusion probabilities, as well as combinations of sample inclusion probabilities for members of either 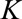 or 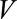.

Test D assessed the robustness of the venue-based–generalized network scale-up method to different rates of reporting error. The venue-based–generalized network scale-up method is robust to false-negative reports,^33^ which occur when members of the frame population fail to report ties to members of the key population. To expand on the previous study,^33^ we tested robustness to false positives (operationalized as the false-positive rate, FPR, see eAppendix D; http://links.lww.com/EDE/B558), which occur when frame population members report knowing more key population members than they in fact do. False-positive reporting is a problem for the generalized network scale-up method.^33^

**FIGURE 4.**
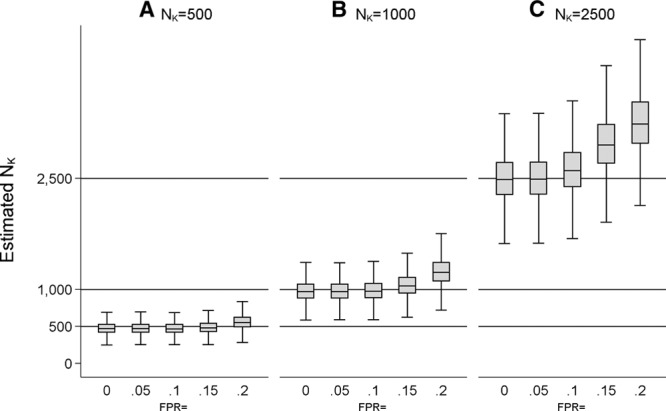
Box and whisker plots of results of test D, which explores variation in the false-positive rate. Note that outlier values are not shown.

Figure 4 presents the test D’s results using the same organization as Figure 2 except that each panel’s *x* axis now indexes increases in false-positive reporting. The results highlight an interesting interaction between false-positive reporting and key population size. For small key populations, increases in false-positive reporting had little effect on estimate quality, but higher levels resulted in overestimation in large key populations. Nonetheless, at moderate levels of false-positive reporting, such as those below 10%, population size estimates were generally accurate in all three population size scenarios. As shown in Table 1, relative biases for the venue-based–generalized network scale-up method were lower than 6.0% and of comparable magnitude to the unbiased scenarios in all scenarios with low false-positive reporting, and only in the largest population size scenarios did relative bias exceed 10% at any levels. These findings indicate that the venue-based–generalized network scale-up method is robust to moderate false-positive reporting.

## DISCUSSION AND CONCLUSIONS

To hasten the end of the HIV/AIDS epidemic, UNAIDS has adopted the “90-90-90” cascade of care targets: 90% diagnosis rates among those living with HIV, 90% antiretroviral treatment rates among those diagnosed, and 90% viral suppression rates among those receiving antiretrovirals.^55^ No country has yet met these goals, although some are close, and diagnoses are the most pressing challenge. Global estimates suggest diagnosis rates are at 54%, treatment rates are at 76%, and viral suppression rates are at 78%.^56^ Targeting outreach programs to key populations may increase diagnosis. Key population sizes vary substantially from place to place, however, and outreach resources are limited. These factors make key population size estimation a critical component of an effective HIV/AIDS response. Not knowing that, for instance, a provincial hub has more key population members than a capital city might thwart optimized outreach and the alignment of resource deployment and demand. As such, new approaches for key population size estimation are of great interest to public health.

We developed a new approach, the venue-based–generalized network scale-up method, for estimating the sizes of key populations that can be applied to a very popular hidden population sampling scheme, venue-based sampling. Our method builds on the previous study focused on estimating population sizes with respondent-driven sampling.^33^ We used a simulation evaluation framework to test the venue-based–generalized network scale-up method’s robustness to commonly experienced issues and found minimal biases and acceptable sampling variability. These results held across ranges of population size, even when surveying only a small number of key population members, and they were robust to different distributions of sample inclusion probabilities among population members and moderate amounts of false-positive reporting. Further study could test different ways of aggregating disparate estimates of the assumed size of the overall venue-attending population and continue to refine methods for obtaining accurate estimates of that quantity. The development of variance estimators for the venue-based–generalized network scale-up method, generalized network scale-up method, and traditional network scale-up methods is also a research area of high priority; there are substantial limitations to current variance estimation approaches in scale-up studies, including those using the generalized network scale-up method.^33^

By leveraging information on the social networks of key population members, the venue-based–generalized network scale-up method arrives at its estimates from a different theoretical angle than other commonly used population size estimation approaches. Our results indicate that the method works in circumstances that are particularly problematic for proportionate scaling methods, such as when few key population members are sampled. In this way, it adds value to venue-based sampling studies where small samples of key population members might preclude reliable size estimation. The venue-based–generalized network scale-up method also allows for estimates even when members of the key population infrequently attend venues (but, note its assumption that all key population members have nonzero probabilities of venue attendance). For some key populations in some contexts, venue-based sampling can be quite limited if most group members infrequently attend venues and only meet one another via. social media.

The venue-based–generalized network scale-up method should not supplant other key population size estimation approaches. Rather, it provides alternate information that can complement and triangulate other data. For instance, researchers could combine the method with commonly used aggregation and proportionate scaling approaches, or with capture-mark-recapture approaches, and Bayesian techniques could help combine across approaches.^57^ Expanding the toolkit of population size estimation procedures for key populations is an important step in the quest to achieve cascade of care targets and hasten the end of the HIV/AIDS epidemic.

## Supplementary Material
