# Supplementary material for: Estimating Hidden Population Sizes with Venue-based Sampling: Extensions of the Generalized Network Scale-up Estimator
Source: Epidemiology. 2019 Sep 30;30(6):901–10. doi: 10.1097/EDE.0000000000001059 (PMC6768707; doi:10.1097/EDE.0000000000001059)
Supplement: Supplementary file 3 [file ede-30-901-s003.docx]

**Supplemental Digital Content**

*eAppendix A: Notation and Assumptions*

We denote the universe of individuals as $U\equiv K\cup V\cup Z$ and note that it comprises three mutually exclusive subsets: $K$ denotes members of the key population of interest (e.g., men who have sex with men, female sex workers, or other groups), all of whom we assume are on the sampling frame; $V$ denotes members of the sampling frame who are not members of the key population; and $Z$ denotes individuals who are not on the sampling frame. Note that the sampling frame, $F\equiv K\cup V$, is the union of $K$ and $V$ and that all members of the key population are assumed to be on the sampling frame, $K\subseteq F$. We use the term hidden population to refer to the general case of hidden populations and the term key population to refer to the specific case of populations at elevated risk of HIV/AIDS. Of interest is knowing the size of the key population, which we define as $N_{K}=|K|$. We define the sizes of other groups in a similar fashion; for instance, $N_{U}=|U|$ is the size of the universe and $N_{F}=|F|$ is the size of the sampling frame. We define the sample sizes using similar notation where, for instance, $n_{K}$ is the number of units sampled from members of $K$.

We refer to a given sample from the sampling frame $F$ as $S_{F}$ and a sample taken exclusively from key population members as $S_{K}$. We distinguish operations conducted over subpopulations from operations conducted over samples of the subpopulation. For instance, $N_{K}=\sum_{j\in K} 1$ is the number of people in the key population $K$ (i.e., the cardinality of $K$), while $n_{k}=\sum_{j\in S_{K}} 1$ is the size of the sample of key population members. In reference to probabilities of inclusion in a sample, we define “population inclusion probabilities,” $\pi_{i}$, where $\pi_{i}>0 \forall i\in F$, $\pi_{i}=0\forall i\notin F$ and $1=\sum_{i\in F} \pi_{i}$, that govern the probability that each case appears in the sample and sum to one at the population level. Note that for convenience we define population inclusion probabilities to sum to one, whereas some studies define population inclusion probabilities to sum to the sample size, which would require division by the sample size in some estimators we employ. We distinguish population inclusion probabilities from “relative inclusion probabilities” (see below), that are defined to sum to one at the sample level, $\varphi_{i}=\frac{\pi_{i}}{\Sigma_{j\in S_{F}}\pi_{j}}$.

We assume that members of the universe are tied in a social network that is represented by the adjacency matrix $A$, where elements $A_{ij}=1$ if person $i$ knows person $j$ and $A_{ij}=0$ if person $i$ does not know person $j$. We refer to a person who reports on a given network connection as the ego and the people about whom the ego is reporting as his or her alters. We represent individual sample members’ reports of their count of outgoing ties to members of a key population as $y_{iK}=\sum_{j\in K} A_{ij}$. We represent the number of outgoing ties from person $i$ to other groups using similar notation, swapping out the second subscript depending on the group in question: for instance, $y_{iF}=\sum_{j\in F} A_{ij}$ would represent the count of person $i$’s ties to members of the sampling frame while $y_{iU}=\sum_{j\in U} A_{ij}$ would represent the count of his or her ties to members of the universe. We represent in-coming ties to sample members using similar subscripting, where, for instance, $v_{iV}$ would be person i’s number of incoming ties from members of $V$. We refer to $v_{iV}$ as the visibility of person $i$ to members of $V$.

*eAppendix B: Assumptions of Traditional and Network Scale-up Methods and the GNSUM*

Traditional network scale-up approaches make three assumptions that are unlikely to hold in realistic field settings.^1–7^ The *first assumption* is the “no barriers” assumption, which assumes that ties are randomly formed in the network and that there are not barriers (or selective mixing) between members of the hidden population and non-hidden members of the universe. The *second assumption* is the “no transmission error” assumption, which maintains that ego respondents in the sample know their alters’ status on attributes of interest. In the cases of key populations, for instance men who have sex with men, the expectation would be that ego respondents are aware of whether or not each of their alters is a man who has sex with men. The *third assumption*, the “no recall error” assumption, deals with reporting. It assumes that ego respondents in the sample can and will accurately recall and report the number of people they know in each queried group on the survey: how many Marthas, police officers, etc. are among their alters. Only when these three stringent assumptions are met does the traditional network scale-up estimator constitute a maximum likelihood estimator of the size of the hidden population of interest.^8^ While a Bayesian framework has been used in some studies to reduce reliance on these assumptions, it can only be applied to simple random samples of the population and requires additional assumptions and data.^4^

The GNSUM replaces the unrealistic assumptions of traditional network scale methods with three less stringent ones. First, it assumes that hidden population members are equivalently visible to members of the frame population and to a researcher-selected group of alters for whom hidden population members will report on their aggregate visibility (“probe alter condition”). GNSUM uses “probe alters” that are representative of the frame population in general because it assumes that the frame population is large and respondents are unlikely to accurately report (or know) how many ties they have to members of the frame population. In our proposed method, described in the main text, probe alters are not required because the frame population, those who attend venues, is comparatively small and ties to it are knowable. Second, GNSUM assumes that hidden population members can accurately report their visibility in aggregate (“accurate aggregate reports about visibility condition”). In our case, even though each sampled key population member may not know exactly how many people he or she knows that attends venues, this assumption implies that the average will be accurate. GNSUM’s third assumption is that frame population members make no false positive reports to members of the hidden population (“no false reports condition”). This condition, that no one who is not in the hidden population would be reported as being so by members of the frame population, is required to justify the use of two samples, one of the frame population and one of the hidden population. In the main text, we address these assumptions as they relate to VBS.

*eAppendix C: An Alternate Approach to Estimating Total Out-Reports*

It is possible to use alternative approaches to estimate the total out-reports from frame members to key population members. For instance, one could use Gile’s successive sampling (SS) estimator,^9^ which is designed for estimating the population mean in cases where sampling is conducted with probability proportionate to size without replacement (PPSWOR). PPSWOR is a well-studied phenomenon in the statistical and survey sampling literatures.^10–14^ It can be shown that RDS is a PPSWOR process.^9^ We argue that VBS has the same features because frequency of venue attendance governs each person’s sample inclusion probability and the process is taken without replacement, meaning that no single person can be sampled for the study more than once. Individuals who attend venues frequently are most likely to be sampled, while individuals who attend them infrequently are less likely to be sampled. Drawing on computer intensive methods and statistical approximations from the geological discovery literature, where oil field finds are assumed to be PPSWOR and the total volume of remaining oil supply is a quantity of great interest, Gile proposes a SS estimator for RDS based on respondent popularity as an approximation of each participant’s relative inclusion probability. For VBS, we suggest that the same approach could be used by substituting frequency of venue attendance as a measure of relative inclusion probabilities. Combining Gile’s SS estimator of the mean outgoing ties from non-key population members on the frame to key population members ($\hat{\mu}_{VK}^{SS}$) with the known size of the frame population that is not in the key population ($N_{V}$; in this case, how many people who are not hidden population members that attend venues), we can estimate the total number of outgoing ties from key population members to frame members as shown in Equation (S1).

$\hat{\tau}_{VK}^{''}=N_{V}\hat{\mu}_{VK}^{SS}$ (S1)

This estimator of the total number of outgoing ties can be applied in Equation (8) in the main text.

*eAppendix D: Simulation Approach and Methods*

Prior work with the GNSUM has demonstrated its robustness to (i) variation in the size of the frame population $F$, (ii) differential network connectivity rates by group, and (iii) variation in the rate of true positives.^1^ We conducted preliminary tests and found that the conclusions reached by Feehan and Salganik with respect to the GNSUM’s robustness to items i, ii, and iii also hold for the VB-GNSUM estimator, so we do not report them here for the sake of brevity. Our simulations are designed to address other issues not previously considered.

*Commonalities between Simulations*

We conduct a series of simulations with populations of $N_{U}=10,000$ individuals; prior work used $N_{U}=5,000$ individuals.^1^ For each combination of simulation parameters, we create 100 random networks, draw 1,000 simulated samples from each of those networks, and estimate the key population size using VB-GNSUM. In all cases, we define the size of sampling frame $N_{F}=4000$, which is a frame fraction of 40%; prior work found robustness as the frame fraction increased from 10% to 100%.^1^ We create the social network linking individuals in the data using a reduced form version of what Feehan and Salganik did. That is, we use the stochastic block model approach to generate ties between people (note that we treat this as a “true” underlying network and test robustness to errors in reporting about its ties in Test D below). In addition to the two parameters described above, the networks are created with the following parameters:

- $N_{K}$ the size of the key population (see Tests A and B, below)
- $a$ the baseline probability of tie formation
- $b$ an adjustment factor that relatively modifies the baseline probability of tie formation when individuals differ on their membership in $V$
- $c$ an adjustment factor that relatively modifies the baseline probability of tie formation when individuals differ on their membership in $K$

The stochastic block model approach creates random ties between individuals in the network with a baseline probability of tie formation, $a$, that is modified depending on whether the individuals in question are or are not in the same “block,” with blocks being defined by membership in either $V$, $K$, or neither ($U∌F$ or $U∌V\cup K$). A baseline probability of $a=0.05$ would, without modification, suggest that each pair of nodes would have a 5% chance of being tied to one another. The first adjustment factor, $b$, modifies the relative probability of tie formation when two individuals differ in their membership in the $V$ block. A first adjustment factor value of $b=0.10$ would suggest that individuals who differ in their membership in $V$, such as if one person were a member of $V$ and the other were a member of $K$, would be 10% as likely to form as if they were both members of $V$. A second adjustment factor value of $c=0.40$ would indicate that individuals who differ in their membership in $K$, such as if one person were not on the sampling frame ($U∌F$ ) and the other were a member of $K$, would be 40% as likely to form as if they were both members of $K$. Thus, ties in the network are formed stochastically with the mixing matrix shown in Equation (S2).

$M=\left[ \begin{matrix} a & abc & ba \\ abc & a & ca \\ ba & ca & a \end{matrix} \right]$ (S2)

In Equation (S2), the rows represent one person’s membership and columns the other person’s and both rows and columns index, respectively in the following order, membership in either $V$, $K$, or $U∌F$. The mixing matrix thus represents the probability of ties given by six non-redundant combinations of nominators and nominees’ attributes. We denote these combinations VV, KK, UU, VK, KV, VU, UV, KU, and UK, where the first letter represents the group membership of the nominator and the second letter represents the membership of the nominee (note that the matrix is symmetric such that three of the nine possible combinations are redundant because VK=KV, VU=UV, and KU=UK). We use values of $a=0.05$, $b=0.40$, and $c=0.10$ throughout the manuscript. Feehan and Salganik (2016) demonstrate robustness to variation in these parameters. This mixing matrix and these parameters suggest the following expected probabilities of tie formation between different groups: $VV, KK, and UU = 0.050$; $VK and KV = 0.05\times0.10\times0.40 = 0.002$; $VU and UV = 0.05\times0.10 = 0.005$; and $KU and UK=0.05\times0.40=0.020$. Of course, different group sizes will create substantial variation in the realized proportion of network ties between these groups.

We hold two additional factors constant between scenarios. First, we hold constant the number of sample members drawn from $V$ at $n_{V}=200$. We choose this value because empirical VBS surveys typically recruit approximately 200 to 250 cases from venues. Because we vary the size of the number of members of K that are sampled in Tests A and B from 5 to 45 individuals, as described below, the total sample size stays within the desired ranges. Second, we hold constant the true positive reporting rate (TPR) at 100%. Note that we distinguish TPR from the false positive reporting rate (FPR), which we do in fact vary. Below, we describe how we model such variation in Test D. Holding the TPR constant at 100% is equivalent to assuming that respondents accurately report on all of their ties to members of the key population (K) or the frame population (F). Salganik and Feehan analytically and by simulation demonstrate the robustness of the generalized network scale up estimator to variation in the TPR.

*Tests A & B: Variation in* $N_{K}$ *and* $n_{K}$

We test the performance of VB-GNSUM as (a) as the size of the key population varies and (b) as the sample size of key population members varies. We examine three different sizes of $N_{K}$: one where there are 500 members of $K$, one where there are 1,000 members of $K$, and one where there are 2,500 members of $K$. These values represent cases where 5%, 10%, and 25% of the universe ($N_{U}=10,000$) being members of the key population. We also vary the sample size of key population members ($n_{K}$) from 5 to 45 in steps of 10. We conduct these tests simultaneously, assessing all $15=3\times5$ combinations of $N_{K}$ and $n_{K}$ parameters in order to determine VB-GNSUM’s robustness to different sampling fractions from $K$. When $N_{K}$ is 2,500 and $n_{K}$ is 5, 0.2% of members of $K$ are sampled. When $N_{K}$ is 500 and $n_{K}$ is 45, 9.0% of members of $K$ are sampled. When larger fractions are sampled, it is likely to be desirable to use Gile’s estimator, which accounts for the without replacement sampling design, as described in eAppendix C.

In Tests A and B, we hold constant the distribution of sample inclusion probabilities using the method described in Scenario 3 in Test C; Test C also describes tests where we consider alternate distributions of sample inclusion probabilities. In Tests A and B, we also hold constant the FPR at 0%; see Test D below for a case where we consider alternate FPRs. We present results of this test in a figure and table that show key metrics as both $N_{K}$ and $n_{K}$ vary simultaneously.

*Test C: Different sample inclusion probability scenarios*

We evaluate the robustness VB-GNSUM (c) to different scenarios governing the sampling probabilities of members of $K$ and $V$. We do this by comparing results across different distributions of sample inclusion probabilities.

We examine four different distributions of sample inclusion probabilities each of which we apply to members of $V$ and members of $K$ for a total of 16 combinations. We test the following distributions of sample inclusion probabilities:

- Scenario 1) Individuals are assigned into one of five classes that represent their likelihood of attending venues and thus being sampled. Individuals in the first class have a 1/365 probability of being sampled; individuals in the second class have a 12/365 probability of being sampled; individuals in the third class have a 52/365 probability of being sampled; individuals in the fourth class have a 156/365 probability of being sampled; and individuals in the fifth class have a 365/365 probability of being sampled. These values correspond to a model where respondents attend venues once per year, once per month, once per week, several times a week, and every day, respectively. Class membership is assigned uniformly at random throughout the population.
- Scenario 2) Individuals are assigned into one of five classes that represent their likelihood of attending venues and thus being sampled. In this scenario, we use the same likelihoods that we used in Scenario 1, but we vary the method by which individuals are assigned into classes. In this scenario, class membership is assigned inverse proportional to degree. We do this by first obtaining the degree distribution of relevant individuals (either members of $V$ or members of $K$), then by grouping respondents into quintiles of that distribution. Respondents in the lowest quintile are assigned the most frequent venue attendance class, then respondents are assigned increasingly frequent venue attendance classes in decreasing order of their quintile in the degree distribution until respondents in the highest quintile are assigned the least frequent venue attendance class.
- Scenario 3) In this scenario, we use the same procedures as above in Scenario 2, but we use skewed quantiles of the degree distribution rather than quintiles. The first quantile contains individuals at and below the tenth percentile of the relevant degree distribution, the second contains individuals above the tenth percentile and up to and including the twenty-fifth percentile, the third contains individuals above the twenty-fifth percentile and up to and including the forty-fifth percentile, the fourth contains individuals above the forty-fifth percentile and up to and including the seventieth percentile, and the fifth contains individuals above and up to and including in the one hundredth percentile.
- Scenario 4) In this scenario, we assign people sample inclusion probabilities that are directly proportional to their popularity by assigning them probabilities equal to one plus their degree.

In this test, we hold constant the FPR at 0%. See Test D below for a case where we consider alternate FPRs. To maintain consistency with Test A, we test these variations in FPR across the same range of values of $N_{K}$ used in that test ($N_{K}=500$, $N_{K}=1,000$, and$N_{K}=2,500$). In this test, we set the values of $N_{K}=1,000$ and $n_{K}=25$, which are the midpoints of the ranges used in Tests A and B.

*Test D: Sensitivity to FPR*

Finally, we test the sensitivity of VB-GNSUM (d) as the fraction of false positive tie reporting increases, which we control with the FPR parameter that we describe below. We model variation in the FPR as follows; the same process could be used to model variation in the TPR. For each respondent, there is a true number of ties (TNT), which is defined by how many network connections they have to members of whatever group of interest, and the reported number of ties (RNT), which is how many they actually report. For instance, $y_{iK}=\sum_{j\in K} A_{ij}$ is the true number of outgoing ties that person $i$ has to members of $K$, while $v_{iF}=\sum_{j\in F} A_{ij}$ is the true number of incoming ties that person $i$ has to members of $F$. In the simulation, we first generate each relevant TNT via the stochastic block model approach described above, then we define the RNT by applying TPR and FPR parameters.

To understand how RNT is generated from TNT and the FPR and TPR parameters, consider the following. Each existing tie can either be reported, in which case it is a true positive (TP), or not reported, in which case it is a false negative (FN). Non-existent ties can also be reported on incorrectly, in which case they are false positives (FP). Non-existent ties that are not reported on (true negatives) do not factor into the relevant calculations we use below. TNT, RNT, TP, FN, and FP are related via Equations (S3-S7).

$RNT=TP+FP$ (S3)

$TNT=TP+FN$ (S4)

$TP=RNT-FP$ (S5)

$TNT=RNT-FP+FN$ (S6)

$RNT=TNT+FP-FN$ (S7)

RNT is affected by both the true positives (TP) and the false positives (FP) that are reported. Thus, if someone in reality knew persons A, B and C but told the research team that they knew persons A and D, their RNT would be 2 because they reported one TP (person A) and one FP (person D). Conversely, a person’s TNT can be defined in terms of the true positives (TP) and false negatives (FN). The other equations are algebraic manipulations of these basic relationships. We define the true positive rate (TPR) as in Equation (S8).

$TPR=TP/TNT$ (S8)

We define the false negative rate (FNR) as in Equation (S9).

$FNR=1-TPR$ (S9)

We define the false positive rate (FPR) as in Equation (S10).

$FPR=FP/RNT$ (S10)

And we define the true negative rate (TNR) as in Equation (S11).

$TNR=1-FPR$ (S11)

In the simulation, the stochastic block modeling procedure created the true network and thus the TNT for each person. We simulate reporting errors by creating RNT by modifying the true number of ties as in Equations (S12-S14).

$RNT=TNT\times TPR+RNT\times FPR$ (S12)

$RNT\times\left( 1-FPR \right)=TNT\times TPR$ (S13)

$RNT=\frac{TNT\times TPR}{1-FPR}$ (S14)

These procedures allow us to use two parameters TPR and FPR and the TNT generated via the stochastic block modeling approach to control the reporting biases. We assume that $0<TPR\leq1$ and $0\leq FPR<1$. We use RNT in all calculations relevant to the production of VB-GNSUM and other estimates.

We test sensitivity of the results to FPR levels of 0% to 20% in 5% increments. Specifically, we test variation in the FPR of reports of ties to key population members ($y_{iK}$) while leaving reports of the visibility of key population members ($v_{iV}$) at the correct levels. Thus, this test represents a case of systematic underreporting of ties to key population members. To maintain consistency with Test A, we test these variations in FPR across the same range of values of $N_{K}$ used in that test ($N_{K}=500$, $N_{K}=1,000$, and$N_{K}=2,500$). In this test, we set the value of $n_{K}=25$, which is midpoint of the range used in Test B and is consistent with the number of key population members sampled with VBS in empirical surveys. In this test, we also hold constant the distribution of sample inclusion probabilities according to the methods in Scenario 3 of Test C for both the sample inclusion probabilities of members of $V$ and members of $K$.

*eAppendix E. References Cited in Supplemental Materials*

1. Feehan DM, Salganik MJ. Generalizing the network scale-up method: a new estimator for the size of hidden populations. *Sociol Methodol*. 2016;46(1):153-186.

2. Killworth PD, McCarty C, Bernard HR, Johnsen EC, Domini J, Shelley GA. Two interpretations of reports of knowledge of subpopulation sizes. *Soc Netw*. 2003;25(2):141-160.

3. Killworth PD, McCarty C, Johnsen EC, Bernard HR, Shelley GA. Investigating the variation of personal network size under unknown error conditions. *Sociol Methods Res*. 2006;35(1):84-112.

4. Maltiel R, Raftery AE, McCormick TH, Baraff AJ. Estimating population size using the network scale up method. *Ann Appl Stat*. 2015;9(3):1247.

5. Shelley GA, Bernard HR, Killworth P, Johnsen E, McCarty C. Who knows your HIV status? What HIV+ patients and their network members know about each other. *Soc Netw*. 1995;17(3):189-217.

6. Shelley GA, Killworth PD, Bernard HR, McCarty C, Johnsen EC, Rice RE. Who knows your HIV status II?: Information propagation within social networks of seropositive people. *Hum Organ*. 2006;65(4):430-444.

7. Zheng T, Salganik MJ, Gelman A. How many people do you know in prison? Using overdispersion in count data to estimate social structure in networks. *J Am Stat Assoc*. 2006;101(474):409-423.

8. Killworth PD, McCarty C, Bernard HR, Shelley GA, Johnsen EC. Estimation of seroprevalence, rape, and homelessness in the United States using a social network approach. *Eval Rev*. 1998;22(2):289-308.

9. Gile KJ. Improved inference for respondent-driven sampling data with application to HIV prevalence estimation. *J Am Stat Assoc*. 2011;106(493).

10. Kochar SC, Korwar R. On random sampling without replacement from a finite population. *Ann Inst Stat Math*. 2001;53(3):631-646.

11. Murthy MN. Ordered and unordered estimators in sampling without replacement. *Sankhyā Indian J Stat 1933-1960*. 1957;18(3/4):379-390.

12. Raj D. Some estimators in sampling with varying probabilities without replacement. *J Am Stat Assoc*. 1956;51(274):269-284.

13. Rao TJ, Sengupta S, Sinha BK. Some order relations between selection and inclusion probabilities for PPSWOR sampling scheme. *Metrika*. 1991;38(1):335-343.

14. Yates F, Grundy PM. Selection without replacement from within strata with probability proportional to size. *J R Stat Soc Ser B Methodol*. 1953:253-261.
